# Drug-tolerant persister cell in cancer: reversibility, microenvironmental interplay, and therapeutic strategies

**DOI:** 10.3389/fphar.2025.1612089

**Published:** 2025-08-14

**Authors:** Haifeng Li, Wenlong Xu, Wenqi Cheng, Guanxiao Yu, Dongmei Tang

**Affiliations:** ^1^ Affiliated Hospital of Qingdao University, Qingdao University, Qingdao, China; ^2^ Qingdao Hospital, Qingdao Municipal Hospital, University of Health and Rehabilitation Sciences, Qingdao, China; ^3^ Department of Anesthesia, Affiliated Hospital of Qingdao University, Qingdao, China

**Keywords:** drug-tolerant persister (DTP) cells, reversible drug resistance, epigenetic and metabolic reprogramming, tumor microenvironment (TME), therapeutic intervention strategies

## Abstract

Drug-tolerant persister (DTP) cells are a subpopulation of cancer cells capable of surviving therapeutic stress through reversible, non-genetic adaptations. These cells contribute to minimal residual disease and eventual tumor relapse. Understanding the mechanisms that govern the entry into and exit from the DTP state—such as epigenetic remodeling, metabolic rewiring, and transcriptional plasticity—reveals actionable vulnerabilities. This article reviews the biological basis of DTP reversibility, outlines the major challenges in targeting these cells, and proposes innovative therapeutic strategies including epigenetic inhibitors, metabolic disruptors, and adaptive dosing regimens. We also highlight the importance of biomarker development and dynamic monitoring. Targeting DTP cells at their reversible stage may prevent permanent resistance, offering a promising avenue to improve treatment durability and patient outcomes in cancer therapy.

## 1 Introduction

The emergence of resistance to anticancer therapies remains one of the most significant challenges limiting long-term clinical outcomes for cancer patients (Marine et al., 2020; Aldea et al., 2021; Ingham et al., 2025). Despite initial promising responses to targeted therapies and chemotherapy, many tumors inevitably recur due to acquired or intrinsic drug resistance (Vasan et al., 2019; Kieإ et al., 2023; Mannan et al., 2025). In recent years, the recognition of DTP cells as a distinct, non-genetically driven state has provided new insights into the dynamics of drug resistance and tumor relapse (Sharma et al., 2010; Mikubo et al., 2021). Clinically, DTP cells have been significantly implicated in various malignancies, notably non-small cell lung cancer (NSCLC), melanoma, colorectal cancer, and breast cancer. In NSCLC, DTP cells mediate resistance to EGFR-targeted therapies such as osimertinib, resulting in tumor recurrence despite initial effective responses (Wang et al., 2020; Emran et al., 2019). Similarly, melanoma frequently exhibits DTP cells after treatment with BRAF/MEK inhibitors, contributing to adaptive resistance and relapse (Shen et al., 2020; Rambow et al., 2018). Colorectal and breast cancers also harbor DTP populations following chemotherapy or targeted therapies, highlighting a broad clinical significance across diverse cancer types (Russo et al., 2024). DTP cells constitute a subset of tumor cells characterized by their capacity to transiently evade cytotoxic or targeted therapies through adaptive, reversible phenotypic changes rather than stable genetic alterations (Dhanyamraju et al., 2022).

Initially described in bacterial populations that survive antibiotic exposure, the concept of persistence highlights a subpopulation of cells exhibiting reversible tolerance without acquiring permanent genetic mutations (Bigger, 1944). Analogously, cancer DTP cells exhibit several hallmark features, including slow cycling or quiescent phenotypes, altered metabolic states, and extensive transcriptomic and epigenetic reprogramming. Critically, the reversible nature of the DTP state allows these cells to re-enter active proliferation and re-establish drug-sensitive populations upon treatment withdrawal (Russo et al., 2024). Thus, the reversible characteristics of DTP cells suggest both a biological vulnerability and a promising therapeutic opportunity.

Despite growing recognition of DTP cells in cancer therapy resistance, the molecular mechanisms governing their reversible state transitions remain poorly understood. The processes governing entry into, maintenance of, and exit from the DTP state are complex and remain only partially understood, involving epigenetic, metabolic, and transcriptional adaptations. This knowledge gap hampers the development of effective therapeutic strategies to eliminate these cells before they acquire stable resistance. In this mini-review, we summarize current insights into the biological basis of DTP reversibility, highlight the challenges in targeting this transient phenotype, and propose novel intervention strategies that exploit the unique vulnerabilities of DTP cells. By targeting this plastic state, we aim to inform approaches that prevent tumor relapse and improve long-term treatment outcomes.

## 2 Biological basis of DTP cell reversibility

**FIGURE 1:**
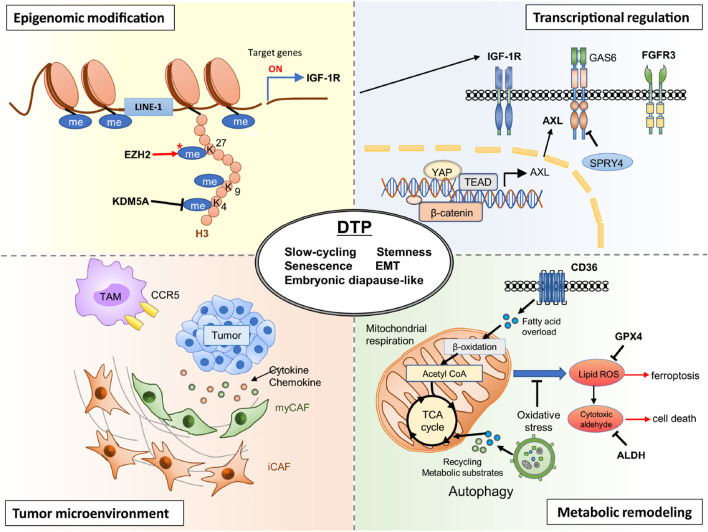
Two proposed models for the generation and evolution of DTP cells. The upper pathway illustrates the pre-existing selection model, where a small subpopulation of DTP-like cells exists prior to treatment and selectively survives upon drug exposure. The lower pathway represents the treatment-induced model, in which drug-sensitive cells acquire a reversible DTP phenotype in response to therapeutic pressure (Sharma et al., 2010; Mikubo et al., 2021). In both models, DTP cells can survive for extended periods under treatment and may later acquire genetic resistance mechanisms (e.g., secondary mutations, bypass pathway activation), ultimately leading to tumor progression or relapse.

DTP cells represent a transient, non-genetic phenotype of cancer cells capable of surviving prolonged drug exposure (Chavez-Dominguez et al., 2023; Izumi et al., 2024). Central to their clinical significance is the remarkable reversibility of this state—DTP cells can exit the drug-tolerant phenotype upon cessation of treatment, reverting back to proliferative, drug-sensitive populations (Sharma et al., 2010; Mikubo et al., 2021). The underlying mechanisms enabling this reversible transition involve complex interplay between epigenetic modulation, transcriptional plasticity, metabolic rewiring, and microenvironmental interactions (illustrated in Figure 1).

### 2.1 Epigenetic regulation and chromatin remodeling

Epigenetic alterations constitute a critical factor driving both the induction and maintenance of the reversible DTP phenotype (Emran et al., 2019). Histone modifications, particularly methylation and acetylation, significantly influence chromatin architecture and gene expression profiles. Sharma et al. first identified histone demethylase KDM5A as essential for the establishment of drug-tolerance in NSCLC (Sharma et al., 2010). KDM5A mediates reversible demethylation of histone H3 lysine 4 (H3K4me), fostering a transcriptionally repressed chromatin state conducive to drug-tolerance. In clinical settings, KDM5A upregulation has been detected in EGFR-mutant NSCLC patient biopsies after EGFR-TKI treatment, and is associated with emergence of a drug-tolerant state (Shaffer et al., 2017). Ongoing clinical evaluations of HDAC inhibitors (such as entinostat) in combination with EGFR inhibitors are underway to overcome such reversible resistance (Chen et al., 2013). In addition, elevated methylation of histone H3 lysine 27 (H3K27me3) by EZH2 further stabilizes this reversible quiescent state by repressing lineage-specific gene expression programs (Marso et al., 2022). Importantly, these histone modifications are dynamic and rapidly reversible upon removal of drug pressure, enabling the swift transition of DTP cells back to drug-sensitive proliferative states.

### 2.2 Transcriptional plasticity and gene regulatory networks

Reversible DTP formation is closely associated with transcriptional rewiring, enabling cells to adapt to therapeutic stress temporarily (Fu and Chen, 2022). Upon drug exposure, cancer cells activate alternative survival pathways including receptor tyrosine kinases (e.g., AXL, IGF-1R), developmental pathways (WNT/β-catenin, YAP/TEAD), and stress-response signaling (STAT3, Aurora kinase A), as indicated in recent studies (Wang et al., 2020; Arasada et al., 2018; Shah et al., 2019; Lee et al., 2014). For instance, in ALK-positive non-small cell lung cancer (NSCLC), treatment with alectinib induces a DTP state characterized by activation of YAP–TEAD and Wnt/β-catenin pathway signaling, contributing to persistence and eventual relapse in patient-derived NSCLC models (Fujimura et al., 2024). Similarly, in colorectal cancer cell lines treated with 5-fluorouracil, a DTP subpopulation shows a diapause-like G_0_/G_1_ arrest and metabolic rewiring that supports survival under cytotoxic stress (He et al., 2024). Taniguchi et al. specifically highlight the induction of AXL signaling through transcriptional upregulation mediated by negative feedback loop disruption involving SPRY4 (Taniguchi et al., 2019). Activation of AXL has been linked to the induction of dormancy and epithelial-to-mesenchymal transition (EMT)-like phenotypes, facilitating survival under drug-induced stress conditions. Additionally, FOXA1-driven upregulation of IGF-1R transcription further exemplifies the reversible transcriptional plasticity utilized by DTP cells to temporarily adapt to drug exposure (Wang et al., 2020). Upon therapy withdrawal, downregulation of these adaptive pathways permits reversion of cells to proliferative, sensitive states.

### 2.3 Metabolic reprogramming as an adaptive response

Reversible metabolic adaptations critically support the persistence phenotype (Mancini et al., 2024). Cancer DTP cells shift their metabolic dependencies from glycolytic pathways toward mitochondrial oxidative phosphorylation (OXPHOS), fatty acid oxidation (FAO), and increased antioxidant capacity, mechanisms that enable survival under therapeutic stress. A phase I clinical trial of the complex I inhibitor IACS-010759 in relapsed/refractory AML and solid tumors has shown preliminary activity against metabolically reprogrammed DTP cells (Yap et al., 2023). Tumor biopsies collected during treatment confirm OXPHOS suppression and reduced ALDH^+^ cell populations (Lu et al., 2021).

Elevated OXPHOS activity not only supports reduced proliferation rates but also limits reactive oxygen species (ROS) accumulation, thereby protecting DTP cells from oxidative stress-induced death (Karki et al., 2022). Increased expression of aldehyde dehydrogenase (ALDH) and glutathione peroxidase 4 (GPX4) further reinforce this adaptive antioxidant response, protecting cells from ferroptosis and lipid peroxidation-induced damage (Raha et al., 2014; Goldman et al., 2019; Hangauer et al., 2017). In BRAF-mutant melanoma, DTP cells emerging after MAPK inhibitor treatment demonstrate increased intracellular calcium signaling via P2X7-mediated ERK reactivation, which supports survival in the drug-tolerant idling state (Stauffer et al., 2024). Additionally, in cisplatin-treated EGFR-mutant lung adenocarcinoma lines, early DTPs downregulate proliferation genes and upregulate lipid-kinase network components such as SOCS1, which has been correlated with poor clinical outcome in treated patients (Chavez-Dominguez et al., 2023). Critically, these metabolic shifts are rapidly reversible—upon drug discontinuation, metabolic profiles revert, reinstating glycolysis-driven proliferation in these formerly quiescent cells.

### 2.4 Influence of the tumor microenvironment (TME)

The reversibility of the DTP state is also regulated by dynamic interactions with the tumor microenvironment. Straussman et al. highlight how cytokines, growth factors, and paracrine signaling within the TME modulate DTP states by influencing intracellular signaling and transcriptional reprogramming (Straussman et al., 2012). For example, hepatocyte growth factor (HGF) produced by tumor-associated fibroblasts (CAFs) and macrophages (TAMs) activates survival pathways, reinforcing temporary drug-tolerance (Heynen et al., 2014; Yano et al., 2008). Similarly, microenvironmental stressors such as hypoxia or nutrient deprivation enhance the persistence phenotype by stabilizing quiescence and survival signaling pathways (Wicks and Semenza, 2022; Poillet-Perez et al., 2021). However, removal of therapeutic stress can rapidly shift microenvironmental conditions, reducing these protective signals and allowing reversion of DTP cells to drug-sensitive phenotypes.

Multiple studies have identified specific genes and signaling pathways underlying the DTP state. For instance, AXL, NGFR, KDM5A, and SOX2 have been linked to reversible persistence through regulation of chromatin remodeling, quiescence, and stress response programs. Additionally, IGF1R, STAT3, and YAP/TAZ signaling are frequently activated in persister cells across NSCLC, melanoma, and breast cancer models, promoting survival and resistance under drug pressure (Sharma et al., 2010; Kurppa et al., 2020). DTP cells also actively reshape the tumor microenvironment (TME) by secreting IL-6, TGF-β, and CXCL12, which recruit immunosuppressive cells (e.g., TAMs, Tregs), thereby reinforcing drug tolerance and promoting tumor recurrence (Oren et al., 2021).

### 2.5 Dynamic entry and exit of DTP states

Collectively, the interplay between epigenetic modifications, transcriptional plasticity, metabolic rewiring, and microenvironmental influences ensures the rapid and reversible nature of the DTP cell state. Entry into the DTP state can be driven by selective expansion of pre-existing subpopulations or induced dynamically in response to therapeutic pressure, reflecting stochastic cell-state transitions. Importantly, as outlined in Figure 1, once the selective drug pressure subsides, these adaptive mechanisms lose their necessity, allowing cells to exit this transient tolerance state and reinitiate proliferation. Understanding these biological bases for DTP reversibility not only provides critical insights into cancer cell plasticity but also highlights novel vulnerabilities that could be therapeutically exploited. Targeting these adaptive, reversible processes may represent an effective strategy to eliminate or substantially delay tumor recurrence and improve long-term treatment outcomes.

## 3 Current challenges in targeting DTP cell reversibility

Despite the promising therapeutic potential offered by exploiting the reversibility of DTP cells, significant challenges currently limit effective targeting and clinical translation of this strategy. These challenges primarily relate to the dynamic complexity of the DTP phenotype, the lack of reliable biomarkers for detecting and monitoring these cells, and practical obstacles encountered in clinical settings.

### 3.1 Complexity and heterogeneity of DTP cell states

A major challenge lies in the inherent complexity and heterogeneity of DTP cells, both within and between tumor populations (Shaffer et al., 2017; Boumahdi and de Sauvage, 2020). Although reversible drug-tolerance is consistently associated with certain hallmark features, such as metabolic rewiring and epigenetic modifications, substantial variability exists regarding the specific pathways activated in individual tumors or even in distinct DTP subpopulations within the same tumor. Such complexity complicates therapeutic targeting since interventions effective against one subset of DTP cells may prove ineffective against another (Rambow et al., 2018). Moreover, cells transitioning between proliferative and quiescent states display distinct molecular vulnerabilities at each stage, necessitating highly precise therapeutic timing and selection of appropriate molecular targets.

### 3.2 Lack of robust and specific biomarkers

Another major impediment is the absence of well-defined biomarkers capable of accurately identifying and tracking DTP cells in clinical samples (Pu et al., 2023). Current detection methods rely primarily on indirect markers such as reduced proliferation rates or elevated expression of certain proteins like KDM5A, ALDH, or AXL. However, none of these markers are uniquely specific to DTP states, and their expression may overlap significantly with other tumor cell phenotypes, such as cancer stem cells or senescent cells. This lack of specificity impedes the ability to monitor the formation, maintenance, and reversion of DTP states during treatment, critically restricting the development of targeted therapeutic approaches.

### 3.3 Translational limitations and clinical challenges

Translating the biological insights gained from preclinical studies of DTP reversibility into effective clinical interventions presents additional practical challenges (Brubaker and Lauffenburger, 2020; Mahalmani et al., 2022). First, continuous, detailed longitudinal sampling of patient tumors required to detect reversible state changes remains difficult due to ethical, technical, and logistical constraints. Second, even with robust biomarker identification, clinical validation demands extensive prospective trials specifically designed to target transient DTP states, which are complicated by the dynamic nature of tumor adaptation during therapy. Finally, clinical implementation of combination therapies or intermittent drug-dosing strategies—potentially beneficial for exploiting DTP reversibility—faces obstacles including increased toxicity, cost, patient adherence issues, and difficulties in precisely timing drug withdrawal or administration intervals.

### 3.4 Risk of inducing stable resistance

An additional critical concern when therapeutically exploiting DTP reversibility is the risk of unintentionally facilitating the transition of persister cells into genetically stable, irreversibly resistant populations (Mieras et al., 2016; Ikai et al., 2013). Studies indicate that prolonged drug exposure may allow DTP cells sufficient time to acquire permanent genetic alterations, such as secondary resistance mutations or genomic amplifications, transitioning from a reversible adaptive state into irreversible resistance (Dhanyamraju et al., 2022). Thus, therapeutic strategies must carefully balance exploiting reversibility without inadvertently driving the selection of genetically resistant subclones.

### 3.5 Limited preclinical and clinical models

Lastly, existing preclinical models inadequately reflect the clinical reality of cancer persistence, limiting their predictive value. Most experimental data on DTP cells derive from *in vitro* cell line studies or short-term animal xenograft models that poorly represent tumor complexity and heterogeneity in patients (Moghal et al., 2023). Such models do not fully capture critical tumor microenvironmental interactions or immune pressures encountered clinically, potentially overestimating or underestimating therapeutic efficacy. Developing more accurate and representative experimental platforms, such as patient-derived organoids or sophisticated immune-competent animal models, is essential for translating laboratory discoveries into effective clinical interventions.

Addressing these diverse challenges through a multidisciplinary approach—integrating precise biomarker development, advanced modeling, carefully designed clinical studies, and cautious therapeutic timing—is paramount to fully exploiting DTP reversibility as a strategy for overcoming therapy resistance.

## 4 Innovative therapeutic intervention strategies

Given the complexity and dynamic nature of DTP cells, conventional monotherapies have proven insufficient for fully eliminating these adaptive populations. Thus, innovative therapeutic strategies aimed explicitly at exploiting the reversible characteristics of DTP cells are urgently needed, as summarized in Table 1. Emerging evidence provides a strong rationale for several promising intervention approaches, including combination therapies targeting epigenetic and metabolic vulnerabilities, intermittent or adaptive dosing strategies, and modulation of the tumor microenvironment.

**TABLE 1:** Summary of therapeutic strategies targeting DTP cells.

Strategy	Target/Vulnerability	Example agents	Cancer types	Mechanism of action	References
Epigenetic Modulation	Histone demethylation, repressive chromatin	KDM5 inhibitors (CPI-455, ryuvidine), HDAC inhibitors	NSCLC, breast cancer	Reverses epigenetic silencing; sensitizes cells to therapy	Vinogradova et al. (2016), Guler et al. (2017)
Metabolic Targeting	Mitochondrial respiration, FAO, antioxidant systems	OXPHOS inhibitors (IACS-010759), FAO inhibitors, GPX4 inhibitors, ALDH inhibitors (disulfiram)	AML, melanoma, lung cancer	Induces metabolic stress or ferroptosis in DTP cells	Yap et al. (2023), Raha et al. (2014)
Intermittent/Adaptive Dosing	Reversibility of drug-tolerance	—	Melanoma, NSCLC	Prevents transition to stable resistance; exploits DTP state plasticity	Hangauer et al. (2017), McGehee and Mori (2024)
Microenvironment Disruption	HGF/MET, IGF-1/IGF-1R, cytokine support	MET inhibitors (crizotinib), IGF-1R inhibitors (linsitinib)	NSCLC, pancreatic cancer	Blocks paracrine survival signals maintaining DTPs	Straussman et al. (2012)
Combination Therapies	Multiple simultaneous vulnerabilities	Epigenetic + targeted drugs; metabolism + chemotherapy	Melanoma, breast cancer, NSCLC	Synergistically eliminates diverse DTP subpopulations	Goldman et al. (2019)
Biomarker-Guided Precision Approaches	DTP-specific gene expression or phenotypic states	Single-cell RNA-seq, ALDH/AXL markers	Various solid tumors	Enables personalized treatment timing and monitoring	Zhuang (2021), Chang et al. (2022)

### 4.1 Epigenetic targeting and chromatin modifying therapies

Epigenetic remodeling plays a pivotal role in driving reversible drug-tolerance phenotypes. Therefore, therapeutic agents targeting key epigenetic regulators represent promising avenues to disrupt DTP establishment and maintenance. For example, inhibitors targeting histone demethylases (such as KDM5 inhibitors like CPI-455 or ryuvidine) have demonstrated efficacy in preclinical models by preventing the reversible repression of chromatin states necessary for DTP survival (Vinogradova et al., 2016; Mitsui et al., 2019). Similarly, histone deacetylase (HDAC) inhibitors such as trichostatin A or entinostat may reverse the transcriptionally repressive states established in DTP cells, restoring sensitivity to initial anticancer treatments (Sharma et al., 2010; Guler et al., 2017). Combining these epigenetic modulators with targeted therapies or chemotherapy could thus synergistically eliminate persister populations, preventing resistance emergence.

### 4.2 Exploiting metabolic vulnerabilities of DTP cells

Metabolic reprogramming observed in DTP cells provides another therapeutic opportunity. As previously described, DTP cells shift toward increased dependence on mitochondrial oxidative phosphorylation (OXPHOS), fatty acid oxidation (FAO), and enhanced antioxidant responses. Targeting these altered metabolic pathways presents a potentially powerful strategy to specifically eradicate DTP cells. For instance, inhibition of mitochondrial OXPHOS through agents such as oligomycin or IACS-010759 significantly reduces persister cell viability in multiple cancer models (Yap et al., 2023; Lu et al., 2021). Additionally, blocking fatty acid oxidation via inhibitors like etomoxir, thioridazine, or ranolazine selectively eliminates DTP subpopulations, capitalizing on their increased reliance on FAO for energy production under drug-induced stress conditions (Redondo-Muأ١ et al., 2023). Furthermore, exploiting antioxidant vulnerabilities through aldehyde dehydrogenase (ALDH) inhibition with agents like disulfiram, or glutathione peroxidase 4 (GPX4) inhibition, can induce ferroptosis specifically in DTP populations, further reducing residual tumor burden (Raha et al., 2014).

### 4.3 Intermittent dosing and adaptive treatment strategies

Intermittent dosing, or “drug holiday” strategies, have emerged as innovative approaches specifically designed to exploit the reversible nature of DTP states (Boshes and Manschreck, 2016; Meredith, , 1996). Continuous therapy often selects for cells transitioning into stable resistance phenotypes; conversely, strategic treatment interruptions can destabilize adaptive processes in persister cells, causing them to revert to drug-sensitive states. Recent preclinical data indicate that intermittent or adaptive dosing schedules significantly prolong therapeutic effectiveness compared to continuous drug administration by disrupting metabolic and epigenetic adaptations critical for DTP survival (Kruk and Schwalbe, 2006; McGehee and Mori, 2024). Optimizing such schedules to carefully balance tumor control while avoiding toxicities remains a vital area for ongoing clinical investigation.

### 4.4 Targeting the tumor microenvironment to disrupt persistence

The TME substantially influences DTP reversibility by providing paracrine support through growth factors, cytokines, and extracellular matrix remodeling. Innovative therapies aimed at disrupting key microenvironmental signals can thus eliminate protective niches essential for DTP survival. For instance, cancer-associated fibroblasts (CAFs) have been shown to secrete hepatocyte growth factor (HGF), which activates MET signaling in drug-tolerant tumor cells. This paracrine signaling contributes to the maintenance of a reversible DTP state after targeted therapy. *In vivo* studies have demonstrated that blocking the HGF-MET axis can resensitize tumor cells to EGFR inhibitors and suppress relapse in lung cancer models (Straussman et al., 2012; Obenauf et al., 2015). These findings underscore the importance of disrupting stromal-derived signaling to eliminate DTP cells and prevent disease recurrence. For instance, targeting hepatocyte growth factor (HGF)-MET or IGF-1/IGF-1R signaling pathways—both critical for maintaining DTP states mediated through tumor-associated fibroblasts (CAFs) and tumor-associated macrophages (TAMs)—has shown promise in preclinical models (Yi et al., 2018). Moreover, interventions aimed at normalizing tumor hypoxia or nutrient stress could further disrupt microenvironment-driven persistence states, rendering DTP populations vulnerable to conventional anticancer agents.

### 4.5 Integrating single-cell technologies and biomarker-guided strategies

The development and incorporation of innovative single-cell sequencing and high-resolution imaging technologies can revolutionize our capacity to identify, monitor, and target DTP populations dynamically (Zhuang, 2021; Chang et al., 2022). Single-cell analyses provide unprecedented insight into the heterogeneous and transient nature of DTP states, enabling identification of novel biomarkers or molecular signatures specific to these reversible phenotypes. Such precision-based approaches allow for real-time monitoring of tumor responses, informing personalized interventions including optimal timing for combination therapies or intermittent dosing strategies. Biomarker-driven clinical trials utilizing adaptive trial designs would facilitate rapid translation of these insights into clinical practice, maximizing the therapeutic potential derived from targeting DTP reversibility.

### 4.6 Combined therapeutic modalities as the way forward

Given the multifaceted nature of DTP biology, it is increasingly evident that single-agent strategies are insufficient. Future therapeutic paradigms will likely require well-designed combinations of epigenetic modifiers, metabolic inhibitors, adaptive dosing schedules, and microenvironment-targeting agents (Furugaki et al., 2025). Rational design of these multidimensional strategies based on deep biological understanding of DTP dynamics, supported by precise biomarkers and cutting-edge analytical tools, holds significant promise for overcoming the formidable challenge of reversible drug-tolerance in cancer.

In summary, innovative therapeutic strategies capitalizing on the reversible vulnerabilities inherent in DTP cells represent a promising frontier in cancer treatment. Through comprehensive targeting of epigenetic, metabolic, microenvironmental, and temporal dimensions of persistence, these approaches offer exciting opportunities to prevent tumor recurrence and improve long-term patient outcomes significantly. In addition to epigenetic and metabolic interventions, recent approaches have explored targeting the tumor microenvironment, inhibiting anti-apoptotic proteins (e.g., BCL2, MCL1), and utilizing immune checkpoint inhibitors in adaptive schedules. Preclinical models also suggest that targeting ferroptosis and inducing oxidative stress may selectively eliminate DTP cells, offering promising avenues for further exploration (Viswanathan et al., 2017).

## 5 Critical controversies and knowledge gaps in the DTP field

Despite significant advancements in understanding drug-tolerant persister (DTP) cells, critical controversies and substantial knowledge gaps persist, hindering effective clinical translation. First, there remains a lack of consensus regarding the precise definition and identification criteria of DTP cells, as various studies utilize different molecular or phenotypic markers (Mikubo et al., 2021; Hangauer et al., 2017). Second, the cellular origins and lineage dynamics of DTP cells are still under debate; it is unclear whether these cells derive from distinct cancer stem cell-like subpopulations or arise from transient adaptations of a broader tumor cell population (Russo et al., 2024). Third, the specific molecular mechanisms that govern reversibility and reactivation of proliferation in DTP cells remain incompletely characterized. Controversy exists, for example, over the precise roles of metabolic rewiring and epigenetic remodeling in these reversible state transitions (Sharma et al., 2010).

Additionally, current research models present significant limitations. Most experimental evidence is derived from simplified *in vitro* systems or short-term animal models that inadequately capture the complexity and heterogeneity of human tumors (Moghal et al., 2023). Further development of patient-derived xenografts, organoids, and immune-competent *in vivo* models is essential. Lastly, there is a notable gap in clinical trials explicitly targeting DTP populations, especially in solid tumors, underscoring an urgent need for clinical validation and biomarker development (Dhanyamraju et al., 2022). Addressing these controversies and knowledge gaps will be critical to fully harnessing the therapeutic potential of targeting DTP cells in cancer treatment.

## 6 Conclusions and future perspectives

Effectively targeting the reversible nature of DTP cells holds great promise for overcoming therapy resistance and preventing tumor relapse. Throughout this review, we have highlighted several core themes essential to understanding and therapeutically exploiting DTP cells: 1) the critical role of epigenetic modifications, such as chromatin remodeling, in governing reversible drug tolerance; 2) the significance of transcriptional plasticity and adaptive metabolic reprogramming enabling DTP survival; 3) the influence of tumor microenvironmental interactions, notably CAF-derived HGF-mediated MET signaling, that sustain the reversible persister state; and 4) emerging therapeutic strategies including epigenetic inhibitors, metabolic disruptors, intermittent dosing approaches, and microenvironmental targeting. Addressing current controversies—such as the definition and cellular origins of DTP cells—and bridging existing knowledge gaps through advanced preclinical models and biomarker-driven clinical trials are pivotal steps forward. Collectively, these insights and approaches provide a comprehensive framework to develop innovative, clinically relevant strategies aimed at eliminating DTP-mediated recurrence, ultimately enhancing treatment durability and improving patient outcomes.

Moving forward, clinical translation will require strategic integration of combination therapies and adaptive dosing approaches, carefully guided by precise biomarker-driven patient stratification and real-time monitoring. Future clinical trials must emphasize flexible designs and longitudinal tumor analyses to validate interventions targeting DTP reversibility. Ultimately, this integrative approach offers the potential to substantially enhance treatment durability and improve patient outcomes in the face of persistent therapeutic challenges. Encouragingly, several early-phase clinical trials have begun exploring therapeutic strategies aimed at eliminating or exploiting DTP cells. For example, ongoing trials are assessing the efficacy of epigenetic modulators (e.g., HDAC inhibitors), metabolic disruptors (e.g., oxidative phosphorylation inhibitors), and intermittent dosing regimens specifically designed to target persister cell populations (ClinicalTrials.gov identifiers: NCT04566133, NCT05321368). Although these studies are still at preliminary stages, their results may provide critical insights and validation for clinically actionable strategies against drug-tolerant persistence in cancer.
